# Cs^+^ Removal from Aqueous Solutions through Adsorption onto Florisil^®^ Impregnated with Trihexyl(tetradecyl)phosphonium Chloride

**DOI:** 10.3390/molecules181012845

**Published:** 2013-10-16

**Authors:** Lavinia Lupa, Adina Negrea, Mihaela Ciopec, Petru Negrea

**Affiliations:** “Politehnica” University of Timisoara, Faculty of Industrial Chemistry and Environmental Engineering Blvd. Vasile Parvan no. 6, Timisoara 300223, Romania; E-Mails: lavinia.lupa@chim.upt.ro (L.L.); adina.negrea@chim.upt.ro (A.N.); petru.negrea@chim.upt.ro (P.N.)

**Keywords:** Cs^+^ adsorption, trihexyl(tetradecyl)phosphonium chloride, Florisil^®^

## Abstract

This research determined the adsorption performance of Florisil^®^ impregnated with trihexyl(tetradecyl)phosphonium chloride (Cyphos IL-101) in the process of Cs^+^ removal from aqueous solutions. The obtained Florisil^®^ impregnated with the studied ionic liquid was characterized through energy dispersive X-ray analysis and Fourier transform infrared spectroscopy in order to verify that the impregnation with the ionic liquid had occurred. The adsorption process has been investigated as a function of pH, solid:liquid ratio, adsorbate concentration, contact time and temperature. The isotherm data was well described by a Langmuir isotherm model. The maximum adsorption capacities of the Florisil^®^ impregnated with the studied ionic liquid was found to be 3.086 mg Cs^+^/g of adsorbent. The results indicated that the adsorption fitted well with the pseudo-second order kinetic model.

## 1. Introduction

In the last decades the attention of researchers was focused on the problem of environmental pollution with radioactive contaminants. Among all radioactive components Cs^+^ is an important radionuclide from several reasons: relatively long-live, high solubility/mobility and strong γ emitting radiation. Furthermore, it can be easily incorporated into terrestrial and aquatic organisms because of its chemical similarity to potassium [1,2,3]. Therefore it should be removed from waste radioactive solutions. One of the most studied methods of radionuclide removal from waste waters is the liquid-liquid extraction using various solvents [4,5,6,7,8]. In recent years ionic liquids (ILs) have gained considerable attention and were investigated as substitutes for the organic solvents normally used in this application due to their advantages: high-thermal stability, non-inflamability and non-volatility [8,9,10,11]. Based on literature survey was observed that the phosphonium ionic liquids, compared to their imidazolium- or pyridinium-based counterparts, present some advantages: improved thermal and chemical stability, unique miscibility behaviour and solvating properties [10,11,12]. Even so they have not been studied so intensely, and were not used for Cs^+^ removal. Trihexyl(tetradecyl)phosphonium bis-2,4,4-trimethylpentylphosphinate has been used in lactic acid separation [13] and metal ions separation [11,14,15,16,17]. Trihexyl(tetradecyl)phosphonium chloride (Cyphos IL-101) has been used as effective extractant for the removal of bismuth, zinc or platinum [18,19,20,21]. We have also focused on the phosphonium-based ionic liquid, because was observed that from the studied ion exchanges those based on phosphonium (like ammonium molybdophosphate) have been intensively investigated and found to be effective for the removal of caesium, but from acidic liquids [22,23]. Therefore in this paper a fairly cheap IL—trihexyl(tetradecyl)phosphonium chloride (Cyphos IL-101)—was used as the separation medium for Cs^+^ removal from aqueous solutions. Due to its commercial availability and low price, Cyphos IL-101 is a favourable extractant for Cs^+^ removal. In the liquid-liquid extraction some drawbacks may arise like the large amount of ionic liquid used, high viscosity leading unfavourably to dissolution and diffusion, difficulties of separation and recovery and low interface area [24,25,26]. In order to overcome these shortcomings and to enhance the separation capacities of the studied IL this was impregnated onto a solid support. Doing this is combined the advantages of the IL with those of the heterogeneous support materials [11,14,17,18,19,20,24,25,26,27,28,29,30,31]. As a support material an inorganic one (Florisil^®^) was used because of its crystalline and well-ordered periodic pore structure which will develop higher resistance to chemical, thermal and radiation degradation and also its well known remarkable selectivity [14,28,31]. In order to determine the adsorption performance of the Florisil^®^ impregnated with Cyphos IL-101 in the process of Cs^+^ removal from aqueous solutions, equilibrium and kinetic studies were used.

## 2. Results and Discussion

### 2.1. Characterization of the Florisil*^®^* Impregnated with IL

**Figure 1:**
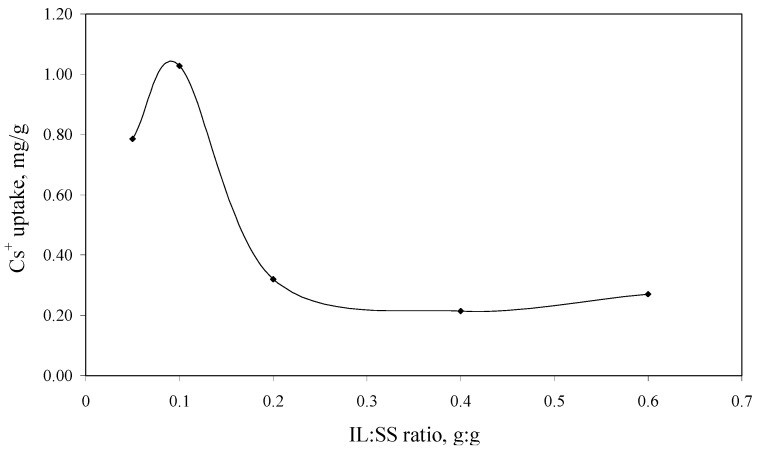
The dependence of the Cs^+^ uptake *versus* of IL:SS ratio (C_i_ = 10 mg/L, V = 25 mL, t = 2 h, pH = 8, m = 0.1 g).

In order to obtain an impregnated solid support with a higher adsorption capacity in the process of Cs^+^ removal from aqueous solutions, the optimum quantity of the studied IL which can be impregnated onto the Florisil^®^ support was determined in the first step. The experimental data regarding the dependence of the Cs^+^ uptake *versus* ratio ionic liquid: solid support (IL:SS) are presented in Figure 1.

Without impregnation with the studied IL the Florisil^®^ support didn’t present any adsorption capacity in the process of Cs^+^ removal from aqueous solutions. It can be observed that by increasing the quantity of the IL impregnated onto the Florisil^®^ support the Cs^+^ ions uptake increases, but for a quantity of IL impregnated onto the solid support higher than 0.1 g the trend is reversed. This happened because at higher quantities of IL, instead of the impregnation of the Florisil^®^ the surface becomes clogged, which leads to the agglomeration of the Florisil^®^ particles, decreasing in this way the surface contact between the adsorbent and adsorbate. The conglomeration of the Florisil^®^ particles impregnated with a higher quantity of IL is visible with the naked eye. The optimum IL:SS ratio used in the further studies is 0.1:1.

**Figure 2:**
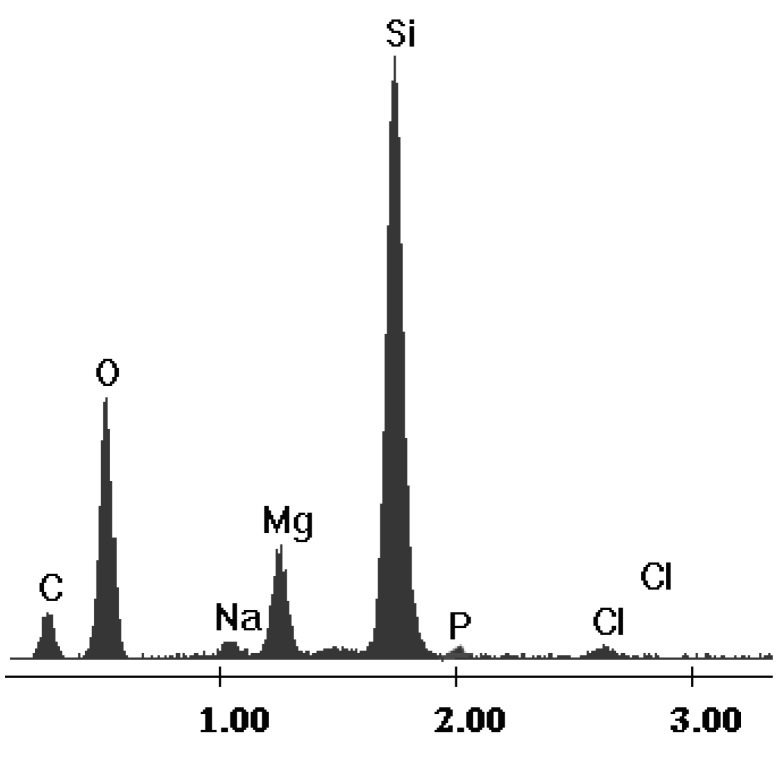
The EDX spectrum of the Florisil^®^ impregnated with IL.

The Florisil^®^ impregnated with the optimum quantity of IL was characterized through energy dispersive X-ray analysis (EDX) in order to demonstrate that the impregnation with IL had occurred. The SEM images of the Florisil^®^ support before and after impregnation with the studied IL are presented elsewhere [32]. The EDX spectrum of the impregnated Florisil^®^ is presented in Figure 2 and in it the characteristic peaks of P and Cl which are components of the ionic liquid can be observed. This analysis thus proved that the impregnation of the Florisil^®^ solid support with the studied IL occurred.

**Figure 3:**
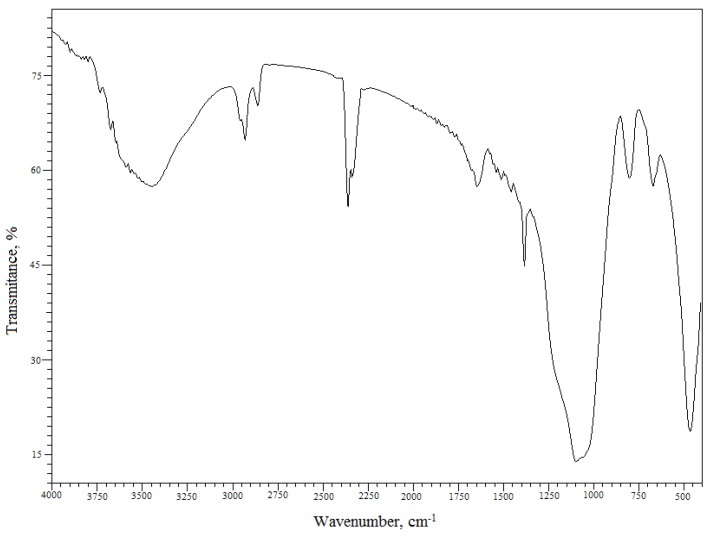
FTIR spectrum of the studied material.

The Florisil^®^ impregnated with IL and used in the process of Cs^+^ removal from aqueous solutions was also characterized by Fourier transform infrared spectroscopy (FTIR). The FTIR spectrum of the Florisil^®^ impregnated with IL after adsorption of Cs^+^ from aqueous solutions is presented in Figure 3 and can be observed that the spectrum is in good agreement with those from the literature [32,33,34,35]. Table 1 shows the characteristic IR bands in [cm^–1^] for the Florisil^®^ impregnated with IL after adsorption of Cs^+^ and the corresponding assignments. The impregnation of the Florisil^®^ with the studied IL is confirmed by the IR peaks around 2,957 cm^−1^, 2,933 cm^−1^, 2,858 cm^−1^ and 1,395 cm^−1^ corresponding to the CH_3_ and CH_2_ stretching vibrations of Cyphos IL 101. The bands at 1,100 cm^−1^, 1,050 cm^−1^ and 800 cm^−1^ are attributed to the group of ν_a_ (C-C) + ν (M-O) + P-C stretching vibrations [33,34,35,36]. The IR spectrum shows an intense and broad band with a maximum at 3,455 cm^−1^, due to the ν(Cs-OH, Cs-OH_2_) vibration, also confirming the retention of Cs^+^. 

**Table 1:** Assignments of the IR vibrations of the studied material [33,34,35].

Wavenumber [cm^–1^]	Assignments
3,455 (s,b)	νOH bonded ν(Cs-OH, Cs-OH_2_)
2,957 (w)	ν_a_ (CH_3_)
2,933 (w)	ν_a_ (CH_2_)
2,858 (w)	ν_s_ (CH_3_)
1,395 (m)	P-C stretching ν_s_ (CH_2_)
1,100 (vs)	P-C stretching ν_a_ (C-C)
1,050 (vs)	P-C stretching ν (M-O)
800 (w)	P-C deformation (out of the plane) ν (M-O)
670 (w)	ν (Si-O)
465 (s)	ν (M-O)

s-strong; v-very; b-broad; m-medium; w-weak; (M = Si, Mg).

### 2.2. pH Influence on the Cs*^+^* Adsorption Process

The experimental data regarding the dependence of the Cs^+^ uptake *versus* the pH of the solution, using an aqueous solution with a Cs^+^ concentration of 10 mg/L and a S:L ratio 0.1 g:0.025 L, under a stirring time of 2 h, was studied. This behaviour is in agreement with other research from the literature [3,4,36]. This is explained by the fact that Cs^+^ is stabilized with the increasing pH (6–8) with the formation of hydroxo complexes ([Cs(OH)_2_(OH_2_)_2....4_]^−^). This is also in agreement with the results obtained at the FTIR analysis. In accord with the experimental data we supposed that Cs^+^ was retained from aqueous solutions through the attraction of the formed negative hydroxo complex of Cs^+^ by the positive phosphonium ions from the IL impregnated onto the Florisil^®^. Therefore for the further experiments we use Cs^+^ solution with an initial pH around value 8.

### 2.3. Influence of the Amount of Impregnated Florisil*^®^* on the Cs^+^ Adsorption Process

**Figure 4:**
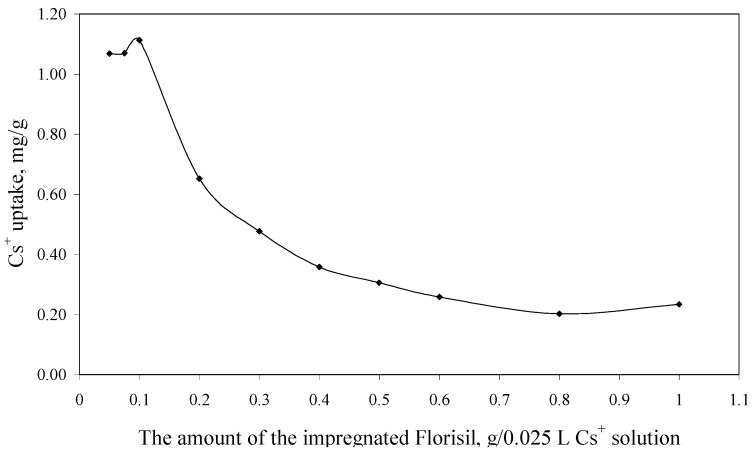
The dependence of the Cs^+^ uptake *versus* the amount of the impregnated Florisil^®^ (C_i_ = 10 mg/L, V = 25 mL, t = 2 h, pH = 8).

The experimental data regarding the dependence of the Cs^+^ uptake *versus* the amount of the impregnated Florisil^®^ used in 0.025 L of Cs^+^ solution, are presented in Figure 4.

From Figure 4 can be observed that an increase in the amount of the impregnated Florisil^®^ used in the process of Cs^+^ removal from aqueous solutions lead to a decrease of the Cs^+^ uptake, because the adsorption capacity is related to the amount of the adsorbent used in the removal process. The optimum S:L ratio used for the further experiments is 0.1 g of impregnated Florisil^®^ for 0.025 L of aqueous Cs^+^ solutions. This parameter is important for the scale up of the studies from the laboratory scale.

### 2.4. Adsorption Isotherm

**Figure 5:**
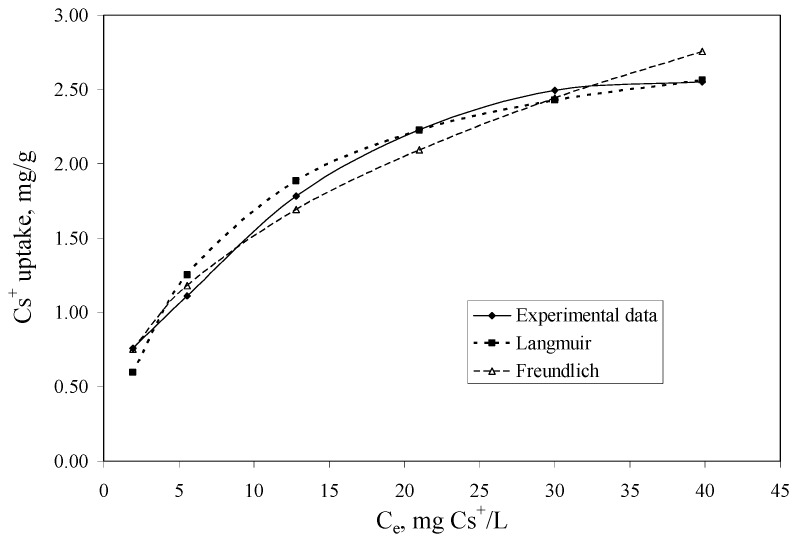
The adsorption isotherms of Cs^+^ onto the impregnated Florisil^®^ with the studied IL (t = 2 h, V = 25 mL, pH = 8, m = 0.1 g, C_i_ = 5–50 mg/L).

The aim of the adsorption isotherm are: (1) to express the affinity of the impregnated Florisil^®^ with the studied IL as a function of its surface properties; (2) to obtain the maximum adsorption capacity of the Florisil^®^ impregnated with the IL in the process of Cs^+^ removal from aqueous solutions and to compare it with other sorbents used in the literature; (3) the obtained results can be used to upscale laboratory batch experiments to a pilot or industrial scale. The equilibrium data were fitted with the Langmuir and Freundlich equations. The isotherms are presented in Figure 5 and the obtained parameters and the correlation coefficients (R^2^) are presented in Table 2.

**Table 2:** Parameters of Langmuir and Freundlich isotherms for Cs^+^ ion adsorption onto Florisil^®^ impregnated with the studied IL.

	Langmuir isotherm		Freundlich isotherm				
*q_m_*_, exp_ mg/g	*K_L_* L/mg	*q_m_*_, calc_ mg/g		*R*^2^	*K_F_* mg/g	*1/n*	*R*^2^
2.6	0.123	3.086		0.9907	0.5658	0.4299	0.9863

The constant *K_F_* can be defined as an adsorption coefficient which represents the quantity of adsorbed metal ions for a unit equilibrium concentration. The slope *1/n* is a measure of the adsorption intensity or surface heterogeneity. For *1/n* = 1, the partition between the two phases is independent on the concentration. The situation *1/n* < 1 is the most common and correspond to a normal L-type Langmuir isotherm, whilst *1/n* > 1 is indicative of a cooperative adsorption which involves strong interactions between the molecules of adsorbate. Values of *1/n* < 1 show favourable adsorption of Cs^+^ ions onto the Florisil^®^ impregnated with IL. Both models showed good correlation coefficients, but those obtained in case of the Langmuir model is more closed to unity, and also the maximum adsorption capacity of the impregnated Florisil^®^ with IL obtained from the Langmuir plot is very close to that obtained experimentally, therefore the experimental data are best described by the Langmuir isotherm suggesting that the adsorption of Cs^+^ from aqueous solution take place onto the homogenous surfaces of the Florisil^®^ impregnated with IL, forming a monolayer on the adsorption surface [1,19]. The adsorption capacities (*q_m_*) of different adsorbents for adsorption of Cs^+^ ions are compared in Table 3. It may be seen that the Florisil^®^ impregnated with Cyphos IL 101 exhibits a good adsorption capacity in the process of Cs^+^ removal from aqueous solutions, compared with other studied materials.

**Table 3:** Adsorption capacity of various adsorbents in the process of Cs^+^ removal from aqueous solutions.

Adsorbent	*q_m_*, mg/g	Reference
Nano-zirconium vanadate	9.1	[3]
Resorcinol-formaldehyde RF	5.56	[37]
Ceiling tiles	0.2128	[38]
Vermiculite	0.646	[39]
Florisil^®^ impregnated with Cyphos IL-101	3.086	Present work

### 2.5. The Influence of Contact Time and Temperature on the Cs*^+^* Adsorption Process and Adsorption Kinetics

**Figure 6:**
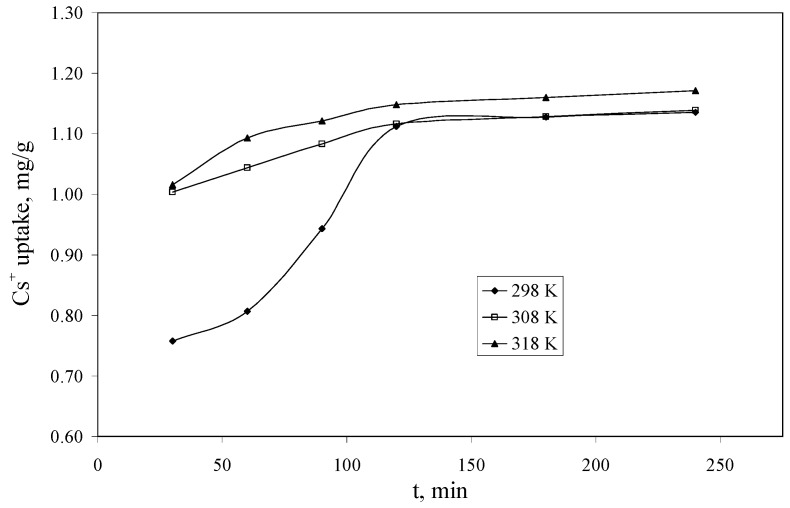
The dependence of the Cs^+^ uptake *versus* the contact time (C_i_ = 10 mg/L, V = 25 mL, pH = 8, m = 0.1 g).

The experimental data regarding the dependence of the Cs^+^ uptake *versus* the contact time, at three temperatures (298, 308 and 318 K) using a Cs^+^ solution having a concentration of 10 mg/L, are presented in Figure 6. From the experimental data can be observed that the equilibrium between Cs^+^ and Florisil^®^ impregnated with IL is achieved after 120 min of contact for all the studied temperatures. This is a very good time comparing with other results from the literature where the equilibrium is achieved in up to 10 days [38]. The increase of the temperature leads to an increase of the quantity of Cs^+^ uptake by the studied adsorbent, but this increase is not significant after the equilibrium is achieved, therefore from the economic point of view is not recommended to increase the temperature.

The kinetics of the adsorption describing the rate of the removal of Cs^+^ is one of the important characteristics that defines the efficiency of adsorption. In order to evaluate the kinetic mechanism that controls the adsorption process, the pseudo-first-order and pseudo-second-order models were tested to interpret the experimental data. Because of the poor regression coefficient (R^2^) values of the Lagergren pseudo-first-order model the results are not included in the text.

**Figure 7:**
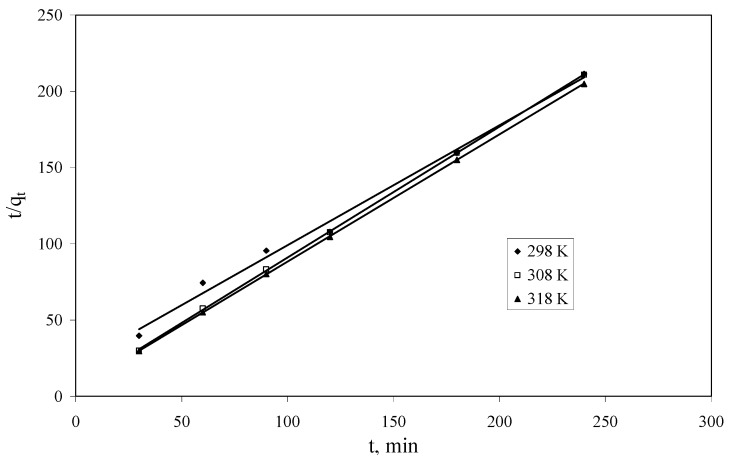
Pseudo-second-order kinetics plots for Cs^+^ adsorption onto Florisil^®^ impregnated with IL.

The pseudo second-order rate constant *k_2_* and *q_e_* were calculated from the slope and intercept of the plots of *t*/*q_t_* *versus* *t* (Figure 7) [1]. The experimental and calculated *q_e_* values, pseudo-second-order rate constants, R^2^ values are presented in Table 4. The calculated *q_e_* values are in agreement with the experimental *q_e_* values and the plots show good linearity, with R^2^ higher than 0.99 for all three studied temperatures. Hence, this study suggested that the pseudo-second-order kinetic model better represent the adsorption kinetics, and the adsorption process has the profile of chemisorptions.

**Table 4:** Pseudo-second-order parameters for Cs^+^ adsorption onto Florisil^®^ impregnated with IL.

Temperature, K	Parameter			
	*q_e_*_, exp_, mg/g	*q_e_*_, calc_, mg/g	*k_2_*, g/(min℘mg)	*R*^2^
298	1.15	1.27	0.0304	0.9926
308	1.15	1.16	0.144	0.9999
318	1.18	1.20	0.146	1

## 3. Experimental

### 3.1. Materials

The trihexyl(tetradecyl)phosphonium chloride (Cyphos IL-101) was purchased from Sigma-Aldrich (Oakville, ON, Canada), and the Florisil^®^ support was supplied by Merck (Darmstadt, Germany). The Cs^+^ aqueous solutions were prepared by adequate dilution of stock solution of 1,000 mg/L (Merck standard solution). In all experiments distilled water was used.

### 3.2. Preparation and Characterization of the Florisil*^®^* Impregnated with IL

The IL was dissolved in acetone (analytical reagent provided by SC Chemical Company SA (Bucuresti, Romania) in a ratio of 0.1 g of IL in 5 mL of acetone. The Florisil^®^ was impregnated with the desired quantity of IL via the dry impregnation method [40]. The EDX analysis of the Florisil^®^ impregnated with IL was investigated by Scanning Electron Microscopy (SEM) using a Quanta FEG 250 instrument (EDAX Inc., Mahwah, NJ, USA), equipped with an Energy Dispersive X-ray quantifier (EDAX ZAF) (EDAX Inc., Mahwah, NJ, USA). The FTIR spectra (KBr pellets) of the studied materials were recorded on a Shimadzu Prestige-21 FTIR spectrophotometer (SHIMADEZU, Tokio, Japan) in the range 4,000–400 cm^-1^.

### 3.3. Adsorption Experiments

The Cs^+^ adsorption from aqueous solutions onto Florisil^®^ impregnated with IL was realised using batch studies. For the batch studies a Julabo SW23 shaker (JULABO Labortechnik GmbH, Sellbach, Germany) with a constant shaking rate was used. To determine the optimum pH the initial pH of the Cs^+^ solutions was adjusted in the 2–12 range using 0.1 N NaOH or 0.1 N HCl, using a S:L ratio of 0.1 g in 0.025 L Cs^+^ solution, keeping the samples under stirring for 2 h. Adsorption isotherms were studied by mixing a known amount of Florisil^®^ impregnated with IL with various intial Cs^+^ concentrations ranging from 5 to 50 mg/L at 298 K and pH = 8. The experimental data obtained were tested with Langmuir and Freundlich isotherms. The effect of contact time was studied varying the time from 30 to 240 min at three temperatures (298 K, 308 K and 318 K). Cs^+^ concentration was determined through atomic emission spectrometry using a Varian SpectrAA 280 type atomic absorption spectrometer (Varian medical systems, Inc., Melbourne, Australia) using an air/acetylene flame. The Cs^+^ uptake was determined using the corresponding mass balance.

## 4. Conclusions

In this work the adsorption properties of Florisil^®^ impregnated with Cyphos Il-101 in the process of Cs^+^ removal from aqueous solutions have been studied. The experiments showed that the adsorption depended on the pH, the maximum desorption capacity being achieved at an initial solution pH of 8. This conclusion is in accordance with the results obtained from the FTIR analysis where was observed that the Cs^+^ ions was retained as hydroxo complexes, which are stable at neutral pH values. The equilibrium data were fitted with the Langmuir and Freundlich isotherm, the best correlaion being obtained by the Langmuir one. The maximum sorption capacity obtained with the Florisil® impregnated with Cyphos IL-101 is 3.086 mg Cs^+^/g of adsorbent. The kinetic studies revealed that the process of Cs^+^ adsorption onto Florisil^®^ impregnated with the studied IL followed the pseudo-second order kinetic model, suggesting the chemisorptions profile of the adsorption process. The proposed method for Cs^+^ removal from aqueous solutions, by using as adsorbent the Florisil^®^ impregnated with Cyphos IL-101, is an efficient one, because it combines the advantages of the ionic liquids with those of the solid support. In this way a smaller amount of the ionic liquid is used for the removal of Cs^+^ from aqueous solutions than in the case of liquid-liquid extraction techniques and there is no risk of loss of the extractant in the aqueous phase. The combined results showed that the obtained new impregnated material represents an efficient adsorbent in the process of Cs^+^ ion removal from aqueous solutions compared with other materials studied in the literature. The experimental results can be easily used to scale up the studies from the laboratory scale.
